# Forecasting the Health Transition and Medical Expenditure of the Future Elderly in China: A Longitudinal Study Based on Markov Chain and Two Part Model

**DOI:** 10.3389/fpubh.2021.774140

**Published:** 2022-01-13

**Authors:** Yuan Gao, Jingbo Li, Xin Yuan

**Affiliations:** ^1^School of Labor Economics, Institute of Population Economics, Capital University of Economics and Business, Beijing, China; ^2^Department of Labor and Social Security, School of Labor Economics, Capital University of Economics and Business, Beijing, China; ^3^Institute of Population and Development, Nankai University, Tianjin, China

**Keywords:** population aging, health status of the elderly, medical expenditure of the elderly, health transition probability, healthy aging

## Abstract

Set in the rapid development of population aging, this study focuses on the relationship between health and medical expenditure of the elderly population. Taking the health and medical expenditure of the elderly as the research object, this study analyzes the characteristics and the intrinsic relationship between them. Based on the future elderly model, this study calculates the transition probability of the elderly's self-assessment health state using the Health Transition Model and estimates the medical expenditure of the elderly by the Two-Part Model. Based on the above, this study predicts the trend of the population size and medical expenditure of the elderly in the next 15 years (2020–2035). Based on the results, the policy suggestions are put forward. To begin with, strengthening health management and health services for the elderly in the construction of healthy China. Next, building a comprehensive system of health care for the elderly in government, society, family, and individual. Then, establishing a long-term care service system as soon as possible. In addition, it is better to establish lifelong health consciousness and cultivate healthy accomplishment behavior. Finally, it is necessary to promote gender mainstreaming in the health field.

## Introduction

Population aging is the irreversible normal social phenomenon, the common future of mankind, and also the fundamental reality of China. Recently, the Political Bureau of the Communist Party of China Central Committee held a meeting to hear a report on major policies and measures to actively respond to an aging population during the 14th Five-Year Plan period. The report pointed out the need to steadily increase the legal retirement age, accelerate the construction of an elderly care service system that integrates family and community institutions and medical care and health care, and implement the three-child policy to actively respond to the aging of the population to provide the necessary guarantee.

According to the seventh national census, there are 260 million people aged 60 or above in China, accounting for 18.7% of the total population. On the basis of the program forecast in the national strategy research on coping with population aging, the aging level will exceed 20% in 2024, reach 30% in 2039, and increase to 34.9% in 2053, when the number of elderly population will reach a peak of 487 million (1). It is a natural rule that the physical function of the elderly will weaken with the increase of age. With the deepening of aging, the scale of the unhealthy elderly population will expand rapidly. According to the Survey on the Living Conditions of the Elderly in Urban and Rural China (2015), the proportion of disabled and semidisabled elderly population in China is 18.3%. Assuming this ratio stays the same, the number of disabled and semidisabled elderly people will approach 100 million by mid-century. There is no doubt that the scale of demand and expenditure for geriatric medical and health services will rise sharply, causing unprecedented pressure on medical and health supply. It is urgent to comprehensively, deeply, and systematically analyze the health status of the elderly and predict the changing trend, so as to provide timely and scientific theoretical basis and information support for the planning and decision-making of the health and economic and social development of the elderly.

At present, the focus of geriatric health research is mainly to explore the influencing factors of geriatric health and the changing trend of geriatric health state. The main factors affecting the health of the elderly can be classified into four categories: First, demographic factors. The elderly in different countries, races, or ethnicities have different evaluations of their own health status. There are significant age and gender differences in the health of the elderly. When using cohort analysis to study the self-assessment of health, it is found that the self-assessment of the health of the elderly decreases with the increase of age (2). Second, social and economic factors. The relationship between health disparities and the socioeconomic status of the elderly is usually discussed using variables such as income (family income and personal income), education level, occupation, and wealth (3–5). Third, health behavior factors. Moderate drinking and exercise are beneficial to health, but heavy drinking and risky drinking increase morbidity and mortality (6). Smoking harms the health of the elderly, and cancer, cardiovascular diseases, and respiratory diseases are highly correlated with smoking (7). BMI is closely related to health, and the higher the BMI, the more prone to coronary heart disease, stroke, ischemic stroke, dyslipidemia, and other diseases (8). The fourth factor is social relations. A good marriage relationship can slow down the decline of health status and reduce the risk of death in the elderly (9). Social participation in the elderly can significantly reduce the risk of death and improve cognitive function in the elderly (10, 11). As for the dynamic research on the health status of the elderly, the current prediction methods of the health status of the elderly include the multistate life table method, the non-covariate method, and the covariate effect method (12–14). Rickayzen and Walsh (2002) were the first to use the Markov method to predict long-term care needs (15).

On the whole, these studies have laid a rich theoretical and methodological foundation for the study of health transfer and future health needs of the elderly. This study focuses on the overall scale analysis of the health state transfer of the elderly and the measurement of the future medical expenditure of the elderly. The main contents include: first, the main research objects of the study are defined, including the health status of the elderly and medical expenses; second, the Health Transition Model (HTM) and the Two-Part Model (TPM) are constructed by using the cohort analysis method; third, quantitative research was conducted on the health status and medical expenditure of the elderly respectively, and the transfer probability of the health status of the elderly and the fitting value of medical expenditure under different health status were estimated; fourth, based on the prediction of the future trend of the number of the elderly population in China, the health probability of different periods is calculated by Markov Chain (MC) to predict the size of the elderly population in different health states. On the basis of this, the total demand and its changing trend of medical and health expenses of the elderly in different health states in the future are judged.

## Methods

### Data Sources

Using the data of China Health and Retirement Longitudinal Study (CHARLS) provided by the National School of Development of Peking University in 2011 and 2013, the elderly people born before 1951 (that is, aged 60 and above) in 2011 were selected as the research object. The sample size of 2011 and 2013 was 17,596 and 18,416, respectively. Based on the longitudinal data, this study matched the sample size of two periods, and finally, 5,487 effective samples were obtained after eliminating the missing and new samples. Considering that the CHARLS data of 2015 does not contain death related data, and in the research framework of this study, the death status of the elderly population needs to be processed as the absorption state, so the latest data of 2015 is not adopted.

### Concept Definition

#### Definition of the Health Status of the Elderly

When evaluating the health of the elderly, the elderly health self-rated was used to calculate the probability of health transition of the elderly. Self-rated health status can not only reflect the individual's health status from both subjective and objective aspects but also evaluate the health status comprehensively. Although self-rated health is essentially an indicator of subjective judgment and has its limitations, its information not only contains the past and future health status of the interviewees but also can be stable and effective for a considerable period of time, with high effectiveness, credibility, and stability. In the CHARLS data, self-rated health questions were divided into two parts, and respondents were randomly assigned to answer questions DA001 or DA002. The questions were: DA001 “How would you say your health?” The answers to this question were “excellent, very good, good, fair, or poor.” DA002 “What do you think of your health?” The answers to this question were “excellent, very good, good, fair, or poor.” Since the respondents were randomly divided into two groups, the two groups of data were combined in this study when dealing with this problem and the data results are relatively robust. Therefore, when dealing with self-rated health variables, two groups of questions related to self-rated health in the CHARLS data were combined and death status was added to obtain six predictable health status indicators, namely, “excellent, very good, good, fair, poor or death,” wherein, “excellent” and “very good” are denoted as health and are represented as state 1. The remaining “good, fair, and poor” were successively expressed as status 2: basic healthy, status 3: unhealthy, status 4: very unhealthy, and status 5: death. Among them, statuses 1, 2, 3, and 4 belong to the transfer state, and 5 is the absorption state. According to the statistics, the distribution of those states is shown in Table 1.

**Table 1:** Estimated value of self-rated health elderly transition probabilities (including death).

	**Percentage (2011)**	**Percentage (2013)**
State 1 (healthy)	12.15	12.96
State 2 (basic heathy)	29.82	30.22
State 3 (unhealthy)	38.49	34.86
State 4 (very unhealthy)	19.54	17.92
State 5 (death)	—	4.05

*Unit: %*.

#### Definition of Medical Expenditure

The data of medical expenditure used in the model are mainly from the microsurvey data of medical expenditure in CHARLS data, including the average medical expenditure of families per year and the average medical expenditure of the elderly individual per month. From the perspective of families, family medical expenditure refers to the total annual medical expenditure (including the part that can be reimbursed by medical insurance) of families with elderly people aged 60 or above, including direct medical expenditure and indirect medical expenditure, excluding health care expenses. Direct medical expenses refer to outpatient, hospitalization, and daily medical expenses of family members; indirect medical expenses refer to the indirect expenses incurred at the time of medical treatment, such as transportation expenses, nutrition expenses, and expenses for accompanying family members.

From the perspective of the individual, the elderly individual medical total expenditure (including medical insurance that can be reimbursed) refers to the medical and health care costs of the elderly, including to go to medical institutions to see outpatient services or accept doorstep medical services (excluding to go to the hospital to do physical examination) costs; the total cost of hospitalization (including the cost paid to the hospital and the cost of the hospital room, excluding the salary of the escort, the cost of transportation for oneself or family members); and the cost of self-treatment (including the cost of purchasing prescription drugs, over-the-counter drugs, traditional Chinese herbal medicines or traditional methods of treatment, taking health supplements such as vitamins and using health equipment). The measurement of the individual medical expenditure of the elderly includes only the direct medical expenditure related to the individual elderly and excludes other expenses related to treatment.

### Model Construction and Methods

#### Model Construction of Health State Transition Probability

Based on the health transition model, logistic regression was used to estimate the health transition probability of the elderly population. The model took the health status of the elderly in 2011 as the basic sample box and analyzed the health status of the same birth cohort in 2013 according to the health status of the elderly aged 60 and above in 2011 with basic demographic characteristic variables, such as economic status variables, health behavior variables, and social relationship variables.

Taking 2011 health state *i* as the independent variable, the model calculates the probability of current health state *j* as follows:


(1) $$P_{i,j,t+2}\left(H_{i,j,t+2}=1|H_{i,t},\;X_{i,t}\right)=\frac{1}{1+e^{-[\alpha_{it}+\beta H_{i,t}+\gamma X_{i,t}+\varepsilon_{it})]}}$$

the odds of the healthy state:


(2) $$\begin{array}{r} \frac{P_{i,j,t+2}\left(H_{i,j,t+2}=1|{H_{i,t},\;X}_{i,t}\right)}{P_{i,j,t+2}\left(H_{i,j,t+2}=0|{H_{i,t},\;X}_{i,t}\right)}=\frac{p_{i,j,t+2}}{1-p_{i,j,t+2}} \end{array}$$

(3) $$\begin{array}{r} =e^{\alpha_{it}+{\beta H}_{i,t},+\gamma X_{i,t}+\varepsilon_{it}} \end{array}$$

The logarithm of odds is taken to obtain the regression model:


(4) $$\begin{array}{r} \ln\left(\frac{p_{i,j,t+2}}{1-p_{i,j,t+2}}\right)=\alpha_{it}+{\beta H}_{i,t}+\gamma X_{i,t}+\varepsilon_{it}, \end{array}$$

(5) $$\begin{array}{r} \;i,j=1,2,3,4;\;t=2011,t+2=2013 \end{array}$$

where, *p*_*i, j, t*+2_ is the probability that the individual is in the healthy state *i* in *t* period and changes to healthy state *j* in *t*+2 period. *H*_*i, t*_ is health state, and covariant variable *X*_*i, t*_ includes demographic characteristics variables (age, gender, and household registration), social and economic status variables (education, medical insurance, and pension), health and behavioral variables (chronic diseases, drinking, smoking, and BMI), and the social relations variables (marital status and social activities).

#### Model Construction of Medical Expenditures Under Different Health Conditions

Whether medical expenditure occurs or not is the optimal decision of self-selection. Therefore, some samples are zero or missing in medical expenditure. If these samples are ignored, the results will be selection bias. To correct the selectivity bias, TPM was used to estimate the medical expenditure of the elderly (16, 17).

The first part of TPM adopts the Probit model to locate whether there is medical expenditure:


(6) $$\begin{array}{r} I_{i}=x_{i}\delta_{1}+\mu_{1i},\;\mu_{1i}\sim N\left(0,\;1\right), \end{array}$$

when I ≥ 0, medical expenditure ME occurs.

The second part is the linear expenditure equation of medical expenditure *ME*_*i*_:


(7) $$\begin{array}{r} Y\left(ME_{i}|I_{i}>0\right)=x_{i\;}\delta_{2}+\mu_{2i},\;\mu_{2i}\sim N\left(0,\;\sigma_{\mu }^{2}\right), \end{array}$$

The likelihood function of this model is:


(8) $$\begin{array}{r} L\left(\delta_{1},\delta_{2},\sigma^{2}\right)=\Pi L_{i}\left(\delta_{1},\delta_{2},\sigma^{2}\;\right), \end{array}$$

where, *L*_*i*_ is the likelihood function of *i* th observation value.

In the empirical analysis, this study established a model equation by predisposing factors, enabling factors, and needing factors for medical services (18, 19).

## Empirical Results and Discussion

### Self-Rated Health Transition Probability of the Elderly

#### Overall

Based on HTM, the health status transition probability matrix of the elderly cohort with different initial health statuses was calculated from 2011 to 2013 (Table 2). The results showed that When the base period is in a healthy state (state 1), the probability of remaining healthy is 0.3211, and the probability of being basic healthy is 0.3561. The probability of deterioration in physical health is 0.2886, of which the probability of transition to unhealthy is 0.2342, the probability of very unhealthy is 0.0544, and the risk of transition to death is 0.0342. When the elderly are in basic health (state 2), the probability of their current health status recovering to health is 0.1459, the probability of maintaining basic health is 0.3707, and the probability of transition to unhealthy or very unhealthy is significantly increased; however, the risk of death is decreased. The probability of returning to the basic health of the elderly who were in an unhealthy state (state 3) is 0.2933. Most of the elderly are still in the original unhealthy state during the current period, and the probability of dying in the current period increases slightly to 0.0413 compared with the previous state. For the elderly who are very unhealthy at the base stage, there is only 0.0441 probability of recovering to health status and 0.3745 probability of recovering to unhealthy status, 1/3 of the elderly continue to maintain very unhealthy status, and the risk of death is 0.0636 at this time.

**Table 2:** Estimated value of self-rated health elderly transition probabilities (including death).

	**State 1**	**State 2**	**State 3**	**State 4**	**State 5**
State 1	0.3211	0.3561	0.2342	0.0544	0.0342
State 2	0.1459	0.3707	0.3492	0.1054	0.0288
State 3	0.1007	0.2933	0.3674	0.1973	0.0413
State 4	0.0441	0.1826	0.3745	0.3352	0.0636
State 5	0	0	0	0	1

The elderly's self-rated health status showed the following transition characteristics: first, no matter what the basic health status of the elderly is, the elderly will mainly keep their original health status until the next period, and the probability of each health status of the elderly to maintain their current status is more than 30%; second, the better the basic health status of the elderly, the lower the probability of the transition to unhealthy or very unhealthy in the next period; third, the worse the basic health status of the elderly, the lower the probability of transition to health and basic health in the next period, and the probability of cross-state physical improvement of the elderly is lower as well. It can be seen that the basal health status of the elderly has an important effect on the health status of the later period.

#### The Age-Specific Transition Probability of the Elderly Self-Rated Health

According to the estimation (Table 3), among the elderly aged 60–64, when they are in a health state (state 1) at the base stage, the probability of remaining healthy at the current stage is 0.3552, the probability of being basic healthy is 0.3271, the probability of being transferred to unhealthy state is 0.2526, the probability of being very unhealthy is 0.0421, and the probability of having died at the current stage is 0.0230. Compared with state 1, elderly in basic health (state 2) at baseline were significantly more likely to transition to poor health and very poor health; however, less likely to die. The probability of returning to basic health is 0.3155 for the elderly in an unhealthy base period (state 3), and the probability of death by the current period is 0.0205, which is similar to state 1. For very unhealthy (state 4) of the elderly, the probability of recovery to health and basic health in the current period is relatively low. The probability of recovery to unhealthy is 0.4141, which is greater than to maintain the original state probability. These results indicate that for people who have just entered the old age, the self-health recovery ability is stronger, and the health status of the young elderly is better on the whole.

**Table 3:** Estimated value of self-rated health elderly transition probabilities by age (including death).

	**State 1**	**State 2**	**State 3**	**State 4**	**State 5**
	**60** **~** **64**				
State 1	0.3552	0.3271	0.2526	0.0421	0.0230
State 2	0.1552	0.3841	0.3610	0.0909	0.0088
State 3	0.1002	0.3155	0.3662	0.1976	0.0205
State 4	0.04	0.1507	0.4141	0.3603	0.0349
State 5	0	0	0	0	1
	**65** **~** **69**				
State 1	0.2757	0.3964	0.2073	0.0657	0.0649
State 2	0.1323	0.3853	0.3667	0.0971	0.0186
State 3	0.0883	0.3212	0.3681	0.2001	0.0223
State 4	0.0546	0.1921	0.3751	0.3334	0.0448
State 5	0	0	0	0	1
	**70** **~** **74**				
State 1	0.3124	0.3048	0.2015	0.1302	0.0511
State 2	0.1328	0.3346	0.3706	0.1209	0.0411
State 3	0.0917	0.2711	0.3815	0.2093	0.0464
State 4	0.0561	0.1931	0.3207	0.3412	0.0889
State 5	0	0	0	0	1
	**75** **~** **79**				
State 1	0.2683	0.3293	0.2317	0.0976	0.0731
State 2	0.1409	0.3264	0.3149	0.1340	0.0838
State 3	0.1011	0.2523	0.3685	0.1721	0.1060
State 4	0.036	0.2432	0.3333	0.3063	0.0812
State 5	0	0	0	0	1
	**Aged 80 and over**				
State 1	0.2947	0.2549	0.3197	0.0717	0.0588
State 2	0.1405	0.3841	0.2222	0.1316	0.1218
State 3	0.1419	0.1768	0.3416	0.1707	0.1689
State 4	0.0752	0.1821	0.2875	0.237	0.2183
State 5	0	0	0	0	1

There was no significant difference between the elderly aged 65–69 years and the elderly aged 60–64 years from the overall health transfer status, but the probability of transition to the basic health of the healthy elderly in the current period increased significantly, and the risk of death of the elderly in each health state also increased slightly.

For the middle aged people aged 70–74 and 75–79, when the base phase is in a healthy state, 1/3 of the elderly will be transferred to basic health. The base period is basic healthy, the probability of recovery to health is low, and most of the elderly maintain the original state or transfer to the next state. When the base stage of the elderly was unhealthy, the probability of the elderly still being unhealthy was 0.3815 and 0.3685, respectively. However, compared with those aged 70–74 years, the risk of overall death was significantly increased in those aged 75–79 years, especially those in basic health and those in poor health.

The elderly aged 80 and above have a lower probability of recovering to a better health state, and the probability of transferring to unhealthy health is higher than that of other age groups.

In conclusion, older people of all ages have a higher probability of maintaining their original health status. The younger elderly are more likely to maintain their current health status or recover to better health status than the older elderly, and the probability of death is slightly lower. In contrast, older adults were slightly less likely to have their baseline health status transitioned to unhealthy or very unhealthy health than younger adults, showing a higher risk of death than other age groups.

### Analysis of Influencing Factors of Medical Expenditure

Table 4 is the regression of the influence of self-rated health of the elderly on the average annual medical expenditure of the family and the average monthly medical expenditure of the elderly according to the two-part model. Model 1 and Model 3 estimate whether the elderly choose to go to hospitals, clinics, or self-care; Model 2 and Model 4 are medical expenditure equations.

**Table 4:** Regression of self-rated health and medical expenditure.

	**Average medical expenditure of families per year**		**Average medical expenditure of the elderly individual per month**	
	**Model 1**	**Model 2**	**Model 3**	**Model 4**
**Self-rated health**	0.192^***^	1,571^***^	0.261^***^	218.8^***^
	(0.0202)	(185.1)	(0.0151)	(31.96)
**Demographic characteristics variables**				
Age	−0.0306^**^	−104.5	−0.0253^**^	17.09
	(0.0156)	(145.9)	(0.0113)	(24.21)
Gender (Female = 0)	0.0181	−88.64	−0.0400	−22.38
	(0.0390)	(356.8)	(0.0292)	(60.46)
Household registration (Non-agricultural = 0)	0.139^***^	−1,401^***^	−0. 149^***^	−256.7^***^
	(0.0523)	(481.4)	(0.0396)	(80.54)
**Social and economic status variables**				
Education (Under Middle School = 0)	−0.00160	129.1	0.0687^*^	36.81
	(0.0531)	(479.7)	(0.0402)	(81.61)
Family income	0.0448^***^	1,185^***^	0.0483^***^	60.50^**^
	(0.0166)	(155.1)	(0.0122)	(26.14)
Pension (No Pension = 0)	0.203^***^	−1,096^*^	0.0420	−199.1^**^
	(0.0577)	(578.0)	(0.0450)	(95.29)
Medical insurance (No Medical Insurance = 0)	0.386^***^	938.3	0.115^*^	263.9^*^
	(0.0834)	(939.1)	(0.0673)	(146.6)
**Social relations variables**				
Marital status (Without Spouse = 0)	0.252^***^	712.7	0.0662^*^	18.01
	(0.0499)	(493.3)	(0.0377)	(79.89)
Social activities (No Attend = 0)	0.0619	−585.9^*^	0.151^***^	−107.9^*^
	(0.0385)	(352.0)	(0.0287)	(60.12)
Constant	−0.341^*^	−8,431^***^	−0.608^***^	−423.8
	(0.204)	(1,984)	(0.151)	(329.4)

^*^, ^**^, and ^***^represent significant at 10, 5 and 1% levels, respectively. The values in brackets are standard deviations.

The elderly family and personal medical participation present the following features: Model 1 and Model 3 show that both family and individual participation in health diagnosis and treatment of the elderly is significant at the level of 10%. The worse the self-rated health status of the elderly is, the more families and individuals choose medical treatment, and the probability of personal medical treatment is higher than that of family medical treatment. When the elderly have a health crisis, family perception lags behind individual perception, and individuals actively respond to health changes through hospitals, clinics, or self-diagnosis. Among the controlling factors that determine whether families and individuals participate in health care, household registration and social security items have the greatest influence. Specifically, first, the older the elderly are, the less likely they are to choose medical treatment, and the more likely families are to provide medical treatment for the elderly than individuals. Second, there are no gender differences in the provision of medical care to older persons by families and individuals. Third, agricultural (rural) families have a higher proportion of family medical diagnosis and treatment probability than nonagricultural (urban) families. Fourth, whether the family provides medical treatment is not related to the education level of the elderly, but the higher the education level of the elderly, the more likely they will choose medical treatment, which is directly related to health awareness, occupation distribution, insurance status, personal income, and other factors. Fifth, the level of family income contributes to the timely provision of medical treatment by both families and individuals, and the level of family income has the same influence on the medical treatment behavior of both families and individuals. Sixth, the elderly participate in endowment insurance and medical insurance to help families to take medical treatment, endowment insurance with regular fixed supplies becomes a part of the individual and family income, the higher the income, the greater the possibility of providing medical treatment.

Family and individual medical expenditures are directly related to the health status of the elderly. Model 2 and Model 4 show that the impact of the self-rated health status of the elderly on both family and individual medical expenditures is significantly correlated at the level of 1%. The worse the self-rated health status of the elderly is, the more family and individual medical expenditures are. The most important individual characteristic that affects the family and individual medical expenditure is the nature of household registration. The influence of the age, gender, and education level of the elderly on the family and individual medical expenditure are not significant at 1, 5, and 10%.

In conclusion, the self-rated health status of the elderly is directly related to whether they receive medical treatment or not and medical expenditure, indicating that the worse the self-rated health status of the elderly, the more they will receive medical treatment, and the higher the amount of family medical expenditure and personal medical expenditure. Differences in individual characteristics of older persons lead to differences in family and individual medical treatment and expenditure. It is worth paying attention to the difference in medical treatment and expenditure caused by the nature of household registration. Nonagricultural household registration has a comparative advantage in the right to enjoy medical resources. In addition, both endowment insurance and medical insurance significantly improve the enthusiasm of the elderly for medical treatment, but the security function of endowment insurance is more direct and timely, and the security of medical insurance has a certain lag, and the advance of medical expenses may reduce the possibility of timely medical treatment of the elderly.

### Calculation of Medical Expenditure for the Elderly

According to the estimated results of the self-rated health status of the elderly, this study estimated the family and individual medical expenditure of the elderly with different self-rated health statuses. The results showed that the average medical expenditure of families with self-rated health was ¥ 3,171.16 per year, the average medical expenditure of families with self-rated basic health was ¥ 3,851.09 per year, and the average medical expenditure of families with self-rated unhealthy increased to more than ¥ 4,000, reaching ¥ 4,579.76 per year (Table 5). The average medical expenditure of families who rated themselves as very unhealthy surged to RMB 7,200.96 per year, which was 1.27 times higher than that of the elderly families who rated themselves as healthy.

**Table 5:** Estimation of medical expenditure for self-rated health elderly.

	**State 1**	**State 2**	**State 3**	**State 4**
Average medical expenditure of families per year (Yuan)	3171.16	3851.09	4579.76	7200.96
Average medical expenditure of the elderly individual per month (Yuan)	177.81	175.73	315.99	647.86

The average medical expenditure of the elderly individual with the self-rated healthy elderly was ¥ 177.81 per month, whereas the average medical expenditure of the elderly individual with the self-rated basic healthy elderly declined to ¥ 175.73 per month, the average medical expenditure of the elderly individual with the self-rated unhealthy elderly was ¥ 315.99 per month, and the average medical expenditure of the elderly individual with the self-rated very unhealthy elderly surged to ¥ 647.86 per month. It is 2.64 times higher than the average medical expenditure of the elderly individual who is self-rated as healthy.

Under the rigid health status of the elderly, assuming that the average monthly personal medical expenditure is stable, according to the estimation results in Table 5, the average annual personal medical expenditure of the elderly who self-rated as very unhealthy, unhealthy, basically healthy, and healthy is ¥ 7774.32, ¥ 3791.88, ¥ 2108.76, and ¥ 2133.72, respectively. The increase in personal health expenditure was higher than that of family health expenditure, and the better the self-rated health status was, the greater the gap between personal health expenditure and family health expenditure was. This reflects that the worse the health status of the elderly is, the more the family medical expenditure for the elderly is, and the medical expenditure for the elderly accounts for more than 50% of the family medical expenditure.

### Age Difference of Medical Expenditure of the Elderly Under Different Self-Rated Health States

The medical expenditure of the elderly in different age groups is measured according to the family, and the individual medical expenditure of the elderly with different self-rated health status output is shown in Table 6. Family and individual medical expenditures of the elderly in different age groups basically support the conclusion that the better the self-rated health status is, the lower the family and individual medical expenditures are. The special feature is that the family expenditure of the 65–69-year-old group is lower than that of the elderly group, and the individual medical expenditure of the 80-year-old and above group is lower than that of the elderly group. The two states of self-rated health and self-rated basic health basically indicate that the physical condition of the elderly may not affect the occurrence of functional conditions, so the contrary decrease in expenditure is consistent with the general understanding.

**Table 6:** Estimation of medical expenditure for self-rated health elderly by age.

	**State 1**	**State 2**	**State 3**	**State 4**
	**60** **~** **64**			
Average medical expenditure of families per year (Yuan)	3010.94	4247.63	4673.69	8801.67
Average medical expenditure of the elderly individual per month (Yuan)	92.21	162.76	308.49	738.63
	**65** **~** **69**			
Average medical expenditure of families per year (Yuan)	4451.52	3728.46	4740.35	6600.16
Average medical expenditure of the elderly individual per month (Yuan)	125.01	152.19	367.76	636.52
	**70** **~** **74**			
Average medical expenditure of families per year (Yuan)	3232.45	3419.31	4925.58	6848.63
Average medical expenditure of the elderly individual per month (Yuan)	88.63	153.79	269.03	618.45
	**75** **~** **79**			
Average medical expenditure of families per year (Yuan)	1662.97	3763.87	4104.32	7135.99
Average medical expenditure of the elderly individual per month (Yuan)	84.36	241.65	354.14	729.68
	**Aged 80 and over**			
Average medical expenditure of families per year (Yuan)	2125.19	3720.39	3855.74	5144.09
Average medical expenditure of the elderly individual per month (Yuan)	355.69	263.43	449.04	589.11

In terms of health status, the elderly aged 65–69 who self-rated as healthy had the highest family medical expenditure of ¥ 4,451.52 per year, followed by the elderly aged 70–74 with an average annual medical expenditure of ¥ 3,232.45. The age group aged 80 and above had the highest individual medical expenditure of ¥ 355.69 per month, followed by the elderly aged 65–69. The average monthly medical expenditure was ¥ 125.01; the family and individual medical expenditure of the elderly showed a “V” shape, and the medical expenditure of the elderly aged 70–74 was less. The elderly aged between 70 and 74 who self-rated as unhealthy had the highest family medical expenditure of ¥ 4925.58 per year, whereas the elderly aged between 70 and 74 had the lowest family medical expenditure of ¥ 3,855.74 per year. The elderly aged between 70 and 74 had the highest individual medical expenditure, and the elderly aged between 70 and 74 had the lowest medical expenditure. The trend of family and individual medical expenditure of the elderly with unhealthy self-evaluation is opposite, which reflects the different attitudes of families and individuals to cope with the unhealthy physical condition of the elderly, and the family medical expenditure lags behind the individual. The family and individual medical expenditure of the elderly who self-rated very unhealthy kept the same trend; the medical expenditure of the elderly aged 60–64 was the highest, and the medical expenditure of the elderly aged 65–74 was the least.

## Forecast of the Health Status of the Elderly and the Total Demand for Medical Expenditure

### Theoretical Basis of Forecast

If *X*_*t*_ = *i*, this process represents the state at time *t* as *i*. Now, assuming that every process is in state *i*, then the probability of getting to the next state *j* is fixed. Therefore, it is assumed that there exists a fixed probability independent of time for:


(9) $$P\{X_{t+1}=j\;|\;X_{t}=i,\;X_{t-1}=i_{t-1},\ldots ,\;X_{1}=i_{1},\;X_{0}$$

(10) $$=i_{0}\}=P_{ij},t\geq 0$$

while *j, i, i*_0_, *i*_1_, …, *i*_t−1_∈*M*, and this random process is called Markov Chain.

Probability represents the probability of moving from a given current state *I* to state *J*, which obviously has:


(11) $$\begin{array}{r} P_{ij}\geq 0,\;i,j\geq 0;\;\overset{\infty }{\underset{j=0}{\sum }}P_{ij}=1,\;i=0,1,\ldots \end{array}$$

A matrix of transition probabilities:


(12) $$\begin{array}{r} P_{ij}=||\begin{array}{cccc} P_{00} & \;P_{01} & \;\cdots & \;P_{0j}\\ P_{10} & \;P_{11} & \;\cdots & \;P_{1j}\\ \vdots & \;\vdots & \;\ddots & \;\vdots \\ P_{i0} & \;P_{i1} & \;\cdots & \;P_{ij} \end{array}|| \end{array}$$

*P*_*ij*_ represents a one-step transfer matrix, or a first-order matrix of a Markov Chain.

Then Chapman-Kolmogorov equation is used to calculate Markov *n*-step transition probability $P_{ij}^{n}$.

Based on the basic principle and the empirical results of the health transition model, the MC first-order matrix of the self-rated health of the elderly was obtained as follows:


(13) $$\begin{array}{r} P_{ij}\left(SAH\right)=||\begin{array}{ccccc} 0.3211 & \;0.3561 & \;0.2342 & \;0.0544 & \;0.0342\\ 0.1459 & \;0.3707 & \;0.3492 & \;0.1054 & \;0.0288\\ 0.1007 & \;0.2933 & \;0.3674 & \;0.1973 & \;0.0413\\ 0.0441 & \;0.1826 & \;0.3745 & \;0.3352 & \;0.0636\\ 0 & \;0 & \;0 & \;0 & \;1 \end{array}|| \end{array}$$

Each element in the matrix represents the probability of the elderly person moving from their initial state of health in the base period to their current state of health. The sum of each row in the matrix is 1.

According to the estimated first-order transition probability matrix of MC, Chapman–Kolmogorov equation was used to simulate the *n*-order transition probability of MC^1^, that is, the health transition probability of the elderly in different cohorts in each t+2 period over time was obtained. Based on the data of the elderly population size predicted by the National Plan of the National Strategy Research Group for Coping with population Aging (2014), 2015 was taken as the starting point for the prediction, and the health self-assessment status of the elderly was analyzed to simulate the annual distribution of disability status of the elderly population from 2015 to 2035.

### Forecast of the Overall Health Status of the Elderly

**Figure 1:**
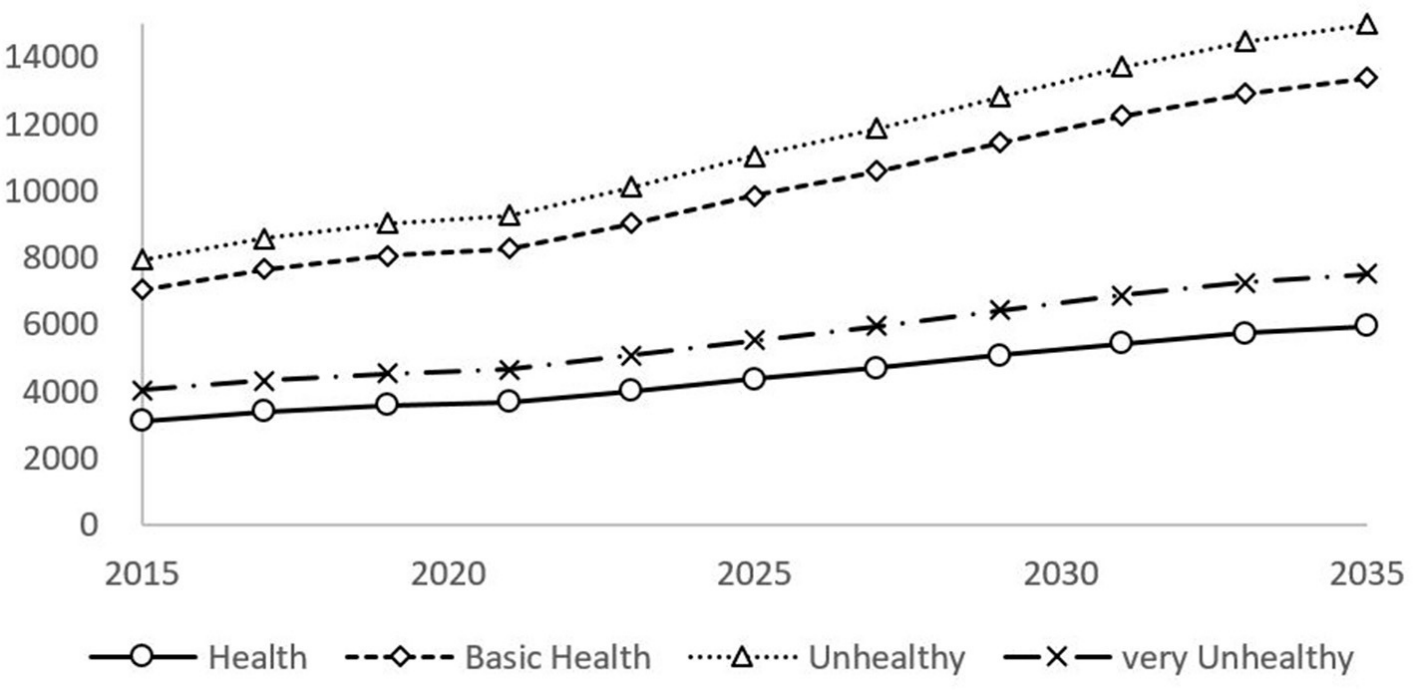
Forecast of the overall health status for SAH elderly in China.

The size of the elderly population with different self-rated health statuses from 2015 to 2035 is shown in Figure 1. The elderly population with an unhealthy state and basic health status is the largest, while the healthy elderly population is the smallest. In addition, the change rate of the unhealthy elderly population is faster than that of other elderly populations, and the change rate of the healthy elderly population is relatively stable. By 2035, the number of healthy, basically healthy, unhealthy, and very unhealthy elderly population will be 59.38 million, 133.78 million, 149.83 million, and 75.09 million, respectively. Compared with 2015, the number of healthy and basically healthy elderly population will increase the most.

### Self-Rated Health Status Prediction of the Elderly in the Different Age Groups

In different age groups (Table 7), from 2015 to 2035, the scale of the healthy, basic healthy, and the unhealthy elderly population has the same consistent trend with age whereas the scale of the very unhealthy elderly population has an inverted V shape with age. In 2015, the number of healthy, basic healthy, unhealthy, and very unhealthy people aged 60–64 is 11.84 million, 24.89 million, 28.36 million, and 13.34 million, respectively. The number of elderly people aged 75–79 in every self-rated health status is the smallest. The scale of the elderly population aged 80 and above who self-rated their health status has rebounded, which confirms the grim trend of the aging population in the future.

**Table 7:** Forecast of health status for SAH elderly in China by age.

	**Healthy**	**Basic healthy**	**Unhealthy**	**Very unhealthy**
	**60** **~** **64**			
2015	1184	2489	2836	1334
2025	1464	3136	3595	1704
2035	1628	3489	3999	1896
	**65** **~** **69**			
2015	686	1853	1968	984
2025	838	2332	2540	1267
2035	1275	3707	4076	1994
	**70** **~** **74**			
2015	477	1070	1318	762
2025	771	1927	2370	1394
2035	1032	2503	3279	1811
	**75** **~** **79**			
2015	361	822	943	506
2025	542	1144	1529	771
2035	711	1434	1977	1112
	**Aged 80 and over**			
2015	446	745	863	465
2025	615	988	1141	596
2035	1102	1791	2024	1067

*Unit: 10,000*.

Toward the middle of the century, the size of the elderly population in every healthy state will rise. By 2035, the number of healthy, basically healthy, unhealthy, and very unhealthy elderly population aged 60–64 will be 16.28 million, 34.89 million, 39.99 million, and 18.96 million, respectively. The number of healthy, basically healthy, and unhealthy elderly population aged 80 and above will all increase compared with the number of elderly population aged 75–79. The number of unhealthy elderly decreased by 4,50,000 from 75 to 79 to 11.02 million, 17.91 million, and 20.24 million, respectively.

### Forecast of Medical Expenditure Demand of the Elderly Under Different Health States

The actuarial model is used to forecast the medical expenditure of the elderly. When forecasting the medical expenditure, it is decomposed into the function of the number of the elderly population in different health states and the per capita medical expenditure in different health states, taking into account the difference of the medical demand of the elderly under different health states. When an individual is in the state *j* at time *t*+2, the average monthly medical expenditure is *ME*_*j*_(*t*+2), *N*(*t*+2) is the number of the elderly population in various health states, and *ME* is the average monthly medical expenditure of the elderly. Assuming that the medical needs and behaviors of the elderly remain unchanged in the next 20 years, the present value of the medical expenditure of the elderly population aged 60 and above is denoted as:


(14) $$\begin{array}{r} ME_{j}\left(t+2\right)=\sum N\left(t+2\right)\cdot \overset{\bar{}}{ME} \end{array}$$

**Figure 2:**
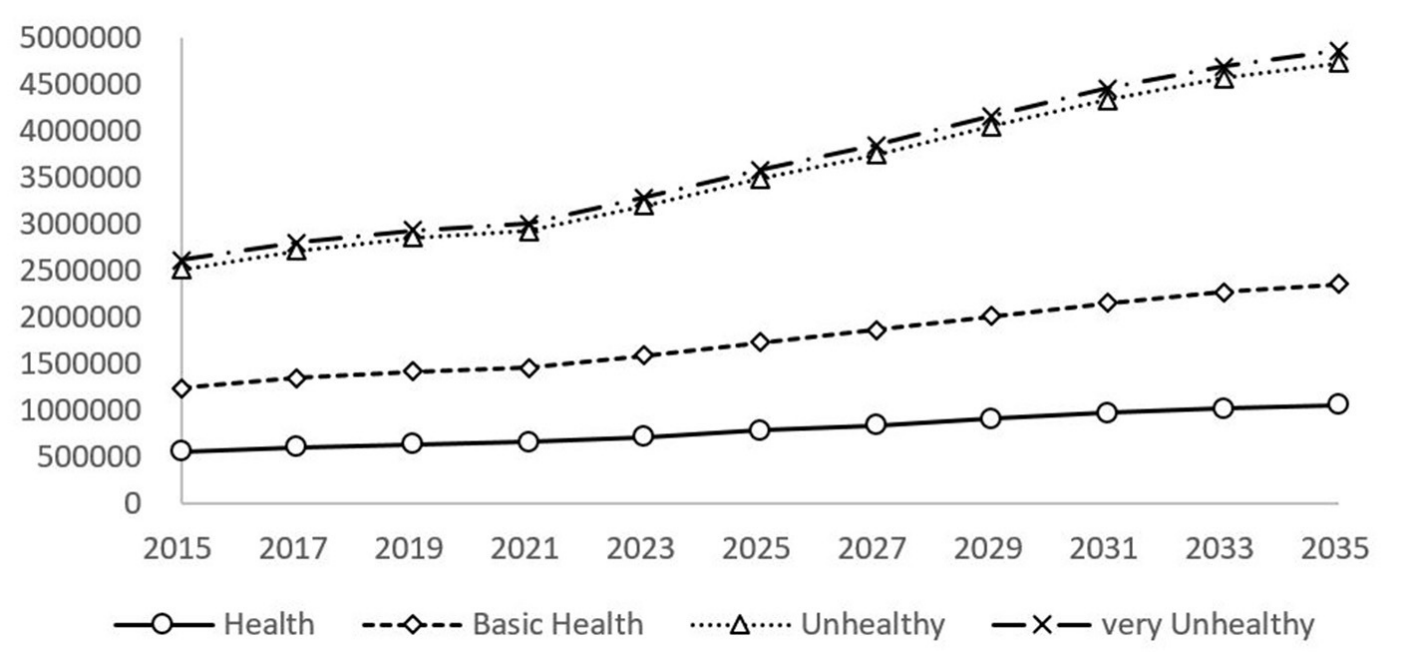
Forecast of annual medical expenditure for SAH elderly in China.

Based on the estimate of the health size of the elderly population, we forecast the medical expenditure of the elderly population under various health conditions (Figure 2). According to the forecast results, the very unhealthy elderly population has the highest level of medical expenditure, and the gap with the unhealthy medical expenditure is small. Relatively speaking, the health and basic health elderly population have a relatively low level of medical expenditure. In 2015, the medical expenditure level of the very unhealthy elderly population was ¥ 26.112 billion, whereas that of the healthy elderly population was only ¥ 5.542 billion, with a difference of ¥ 20.57 billion between the two. In 2035, the gap will reach ¥ 38.088 billion. In 2035, the expenditure on medical care for the elderly in good health, basic health, unhealthy and very unhealthy will be ¥ 10.558 billion, ¥ 23.510 billion, ¥ 47.344 billion, and ¥ 48.645 billion, respectively.

With the rapid development of the aging population in China, the total elderly population is increasing rapidly, and the scale of the disabled elderly population is gradually expanding, which directly leads to the rapid expansion of medical security fund demand. In 2000, the medical expenditure of the elderly population in China accounted for 0.48% of GDP, and in 2015, it accounted for 1.24%, and it is estimated that this proportion will continue to expand. The pressure on the scale of medical expenses for the elderly concerns the health and quality of life of all citizens, the people's livelihood and the health of the people. It has fundamentally changed the intergenerational distribution pattern of medical and health resources, caused potential social intergenerational conflicts and conflicts of interest, and profoundly affected the realization of the strategic goal of Healthy China.

**Figure 3:**
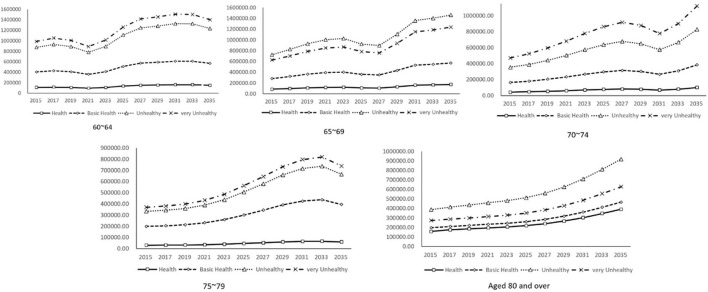
Forecast of annual medical expenditure for SAH elderly in China by age.

The elderly population aged 60–64, 70–74, and 75–79 years old had the largest scale of unhealthy medical expenditure, the elderly population aged 65–69 and 80 years and above had the largest scale of unhealthy medical expenditure, and the elderly population in different age groups had the least health medical expenditure (Figure 3). In 2015, the medical expenditure of the elderly population aged 60–64, 65–69, 70–74, 75–79, 80 and above were ¥ 1.092 billion, ¥ 857 million, ¥ 423 million, ¥ 304 million, and ¥ 1.585 billion, respectively. The elderly population and the young elderly population had the highest medical expenditure. The underage elderly population is a potential human capital resource, and large-scale medical expenditure can effectively improve their health status, which provides conditions for delaying the retirement age. In 2015, the medical expenditure of the elderly population aged 60–64, 65–69, 70–74, 75–79, 80 and above in very unhealthy state was ¥ 9.853 billion, ¥ 6.262 billion, ¥ 4.713 billion, ¥ 3.695 billion, and ¥ 2.738 billion, respectively, showing that the older the population is, spending on health care to deal with very unhealthy conditions is less but still higher than spending on health care for the healthy elderly population. In 2035, the medical expenditure of the elderly population of all ages to deal with very unhealthy physical conditions will be ¥ 14.07 billion, ¥ 12.373 billion, ¥ 11.20 billion,¥ 7.387 billion, and ¥ 6.288 billion, respectively. Compared with 2015, medical expenditure increased by ¥ 4.154 billion, ¥ 6.111 billion, ¥ 6.487 billion, ¥ 3.692 billion, and ¥ 3.55 billion, respectively. The elderly population aged 70–74 and 65–69 had the largest increase in medical expenditure.

## Conclusion

Population aging is an irreversible normal social phenomenon, the common future of mankind, and also the fundamental reality of our country. The physical function of the elderly weakens with age and the dependence on health resources and services is natural. With the rapid development of population aging, the scale of unhealthy elderly people in China is expanding, the demand for health services is increasing rapidly, and the expenditure of medical and health care is increasing markedly. The aging population has exacerbated the challenge and pressure of health and medical expenditure inflation in the elderly population. Paying attention to the health of the elderly and realizing healthy aging is the key to alleviating the pressure of population aging, and also the basic intention of China's active response to population aging strategy in the new era.

Set in the rapid development of population aging, this study focuses on the relationship between health and medical expenditure of the elderly population. Taking the health and medical expenditure of the elderly as the research object, this study analyzes the characteristics and the intrinsic relationship between them. Based on the future elderly model, this study calculates the transition probability of the elderly's self-assessment health state using the health transition model and estimates the medical expenditure of the elderly by the two-part model. Based on the above, this study predicts the trend of the population size and medical expenditure of the elderly in the next 15 years (2020–2035). The main conclusions are as follows:

First, due to the rigid “ratchet effect” of the health status of the elderly population and the characteristics of the marginal decline of medical expenditure, it is decided that the basal period medical expenditure has a significant negative correlation with the health status of the elderly population.

Second, based on the health transition model, it is found that the health changes of the elderly population are mainly affected by initial health, age, and gender. In different initial states, regardless of the health status of the elderly in the base period, continuing to the next stage, the elderly population is primarily in keeping with the original health status, and the probability of maintaining the status quo in the next phase of the elderly population is above 30% percent for each health condition. Among the elderly population, the probability of maintaining an existing state of health or recovering to a better level of health at a younger age is higher than that at an older age, and with a slightly lower death rate. When the elderly are in a state of health in the basal line, in the next period, the health transition probability of women is significantly greater than that of men, whereas the older men have a stronger ability to improve their health status than women.

Third, the health status of the elderly population is directly related to family, personal medical treatment, and medical expenditure. On the one hand, the poorer the health status of the elderly, the higher the rate of medical treatment and medical expenditure. On the other hand, differences in individual characteristics of the elderly population lead to differences in medical treatment and medical expenditure between families and individuals. The poorer the health of the elderly, the more family and personal medical expenses, and personal medical expenditure accounts for more than 50% of the family's medical expenditure. In addition, the medical treatment and medical expenditure of the elderly population of different age groups basically support that the better the self-rated health status, the lower the family and personal medical expenditure.

Fourth, based on the Markov chain and Chapman-Kolmogorov equation, this study forecasts the health status and corresponding medical expenditure of the elderly population in the whole group and the age group during 2020 and 2035. It turns out that in terms of quantity, unhealthy status and basic health status of the elderly population is the largest, and relatively, the size of health status is smallest. In terms of age, the growth trend of the elderly population with healthy, basic health, and unhealthy status is the same with an increase in age, and the growth trend of the unhealthy elderly population is an “inverted V” with age change. In terms of gender, there is no obvious gender difference in the trend of health change among the elderly. In terms of medical expenditure, the elderly population with a very unhealthy status has the highest level of medical expenditure, whereas the mild disability elderly population has the highest medical expenditure.

Based on all the above, the policy suggestions are put forward. To begin with, to strengthen health management and health services for the elderly in the construction of healthy China. Next, building a comprehensive system of health care for the elderly in government, society, family, and individual. Then, establishing a long-term care service system as soon as possible. In addition, it is better to establish lifelong health consciousness and cultivate healthy accomplishment behavior. Finally, it is necessary to promote gender mainstreaming in the health field.

## Data Availability Statement

The raw data supporting the conclusions of this article will be made available by the authors, without undue reservation.

## Author Contributions

YG specialism is Aging Population and Population Economics and Health Economics. JL specialism is Population Economics and Labor Economics. XY specialism is Aging Population and Population Economics. All authors contributed to the article and approved the submitted version.

## Funding

This study was supported by the National Social Science Fund of China [grant number 20CRK014].

## Conflict of Interest

The authors declare that the research was conducted in the absence of any commercial or financial relationships that could be construed as a potential conflict of interest.

## Publisher's Note

All claims expressed in this article are solely those of the authors and do not necessarily represent those of their affiliated organizations, or those of the publisher, the editors and the reviewers. Any product that may be evaluated in this article, or claim that may be made by its manufacturer, is not guaranteed or endorsed by the publisher.

## Footnotes

^1^If more information of Markov process and Chapman-Kolmogorov equation are needed, please contact the author for details.
